# Safety and efficacy of artemether-lumefantrine against uncomplicated *Plasmodium falciparum* malaria during pregnancy: a systematic review

**DOI:** 10.1186/1475-2875-11-S1-O39

**Published:** 2012-10-15

**Authors:** Christine Manyando, Kassoum Kayentao, Umberto D’Alessandro, Henrietta U Okafor, Elizabeth A Juma, Kamal Harried

**Affiliations:** 1 Public Health Department, Tropical Diseases Research Centre, P.O. Box 71769, Ndola, Zambia; 2 Malaria Research and Training Centre, University of Bamako, P.O. Box 1805, Bamako, Mali; 3 Medical Research Council Unit, P.O. Box273, Fajara, The Gambia; 4 Department of Pediatrics, College of Medicine, University of Nigeria, P.O. Box 3295, Enugu, Nigeria; 5 Kenya Medical Research Institute, P.O. Box 54, Kisumu, Kenya; 6 Novartis Pharmaceuticals Corporation, East Hanover, NJ07936-1080, USA

## Background

Malaria during pregnancy, especially *Plasmodium falciparum* malaria, is linked to increased morbidity and mortality, which must be reduced by preventive measures and effective case management [1-3]. The World Health Organization (WHO) recommends artemisinin-based combination therapy (ACT) to treat uncomplicated *P. falciparum* malaria during the second and third trimesters of pregnancy, and quinine plus clindamycin during the first trimester [4]. However, the national policies of many African countries currently recommend quinine throughout pregnancy. Therefore, the objective is to provide a summary of available data on the safety and efficacy of artemether-lumefantrine (AL) in pregnancy.

## Materials and methods

A systematic English-language research identified 16 publications from 1989 to October 2011 with reports of artemether or AL exposure in pregnancy, including randomized clinical trials, observational studies, and systematic reviews.

## Results

Overall, there were 1,103 reports of AL use in pregnant women: 890 second/third trimester exposures; 212 first trimester exposures; and 1 case where the trimester of exposure was not reported. In the second and third trimesters, AL was not associated with increased adverse pregnancy outcomes compared with quinine or sulphadoxine-pyrimethamine, showed improved tolerability relative to quinine, and its efficacy was non-inferior to quinine. Although, few reports suggest that the pharmacokinetics of anti-malarial drugs may change in pregnancy, the majority of studies reported high cure rates and adequate tolerability. As there are fewer reports of AL safety in the first trimester, additional data are required to assess the potential to use AL in the first trimester.

## Conclusions

These findings reinforce the WHO recommendation to treat uncomplicated *P. falciparum* malaria with quinine plus clindamycin in early pregnancy and ACT in later pregnancy.
